# Effects of Annealing Ambient on the Characteristics of LaAlO_3_ Films Grown by Atomic Layer Deposition

**DOI:** 10.1186/s11671-017-1889-z

**Published:** 2017-02-10

**Authors:** Lu Zhao, Hong-xia Liu, Xing Wang, Chen-xi Fei, Xing-yao Feng, Yong-te Wang

**Affiliations:** Key Laboratory for Wide Band Gap Semiconductor Materials and Devices of Education, School of Microelectronics, Xidian University, Xi’an, 710071 China

**Keywords:** LaAlO_3_, ALD, RTA, Interfacial property, Electrical property

## Abstract

We investigated the effects of different annealing ambients on the physical and electrical properties of LaAlO_3_ films grown by atomic layer deposition. Post-grown rapid thermal annealing (RTA) was carried out at 600 °C for 1 min in vacuum, N_2_, and O_2_, respectively. It was found that the chemical bonding states at the interfacial layers (ILs) between LaAlO_3_ films and Si substrate were affected by the different annealing ambients. The formation of IL was enhanced during the RTA process, resulting in the decrease of accumulation capacitance, especially in O_2_ ambient. Furthermore, based on the capacitance-voltage characteristics of LaAlO_3_/Si MIS capacitors, positive *V*_FB_ shifting tendency could be observed, indicating the decrease of positive oxide charges. Meanwhile, both trapped charge density and interface trap density showed decreased trends after annealing treatments. In addition, RTA process in various gaseous ambients can reduce the gate leakage current due to the enhancement of valence band offset and the reduction of defects in the LaAlO_3_/Si structure in varying degrees.

## Background

According to Moore’s law, gate dielectrics applied in complementary metal oxide semiconductor (CMOS) devices with an equivalent oxide thickness (EOT) of no more than 1 nm are needed since the 45-nm technology node. Consequently, insulating materials with much higher dielectric constant than that of silicon oxide or oxynitrides are required to gain an acceptable gate leakage current and static power consumption [1]. Due to its appreciably high dielectric constant (20 ~ 25), large band gap (E_g_ > 5 eV), and valence band offset (VBO > 1 eV) relative to silicon, lanthanum aluminate (LaAlO_3_) has been considered as one of the alternative materials to replace SiO_2_ as the insulator [2, 3]. Benefit from its growth mechanism controlled by a self-limited surface reaction, atomic layer deposition (ALD) is being considered as a promising deposition technique to produce high quality high-k thin films with excellent conformality and precise thickness controllability [4]. However, ALD is a low-temperature deposition technique, thus high temperature post-deposition annealing (PDA) is needed to eliminate trapped charges and dangling bonds in high-k dielectric films after the deposition process [5, 6]. In addition, the annealing treatment can also be of help to reduce interface trap density (*D*_it_) at the insulator/semiconductor interfaces [7]. Unfortunately, PDA process can significantly increase the thickness of interfacial layer (IL) between high-k dielectric and Si substrate by the interdiffusion of the dielectric and silicon, resulting in the decrease of dielectric constant for insulators [8]. Besides, it has been reported that different annealing treatments affect high-k films and interfaces both structurally and electrically in varying degrees [9]. The interfacial properties, including the amount of oxide-trapped charges, fixed oxide charges, interface traps, oxygen vacancies, and dangling bonds, play an important role in determining the electrical characteristics of dielectric film [10].

In this paper, the effects of different annealing ambients on the physical and electrical characteristics of LaAlO_3_ films grown on p-type Si substrate by ALD technique were investigated. Post-grown rapid thermal annealing was carried out at 600 °C for 1 min in vacuum, N_2_, and O_2_, respectively. Among, attentions were focused on the interfacial properties of LaAlO_3_/Si structures to analyze the effects of different annealing ambients.

## Methods

LaAlO_3_ dielectric films were deposited on p-type Si (100) wafers by the Picosun R-150 atomic layer deposition reactor. Prior to the deposition, the wafers were treated with a 1:50 diluted HF solution to remove the native SiO_2_, followed by a 60-s rinse in demonized water. Under the deposition temperature of 300 °C, La(^i−^PrCp)_3_ and TMA were used as the La and Al precursors, while O_3_ was used as the oxygen source. Setting the pulse ratio of La and Al precursor as 3:1, the La:Al stoichiometric ratio of the deposited films is approximately 1:1 [11]. By varying the number of ALD cycles, LaAlO_3_ films with the thickness of ~4 and ~10 nm were prepared. After the deposition of LaAlO_3_ films, post-grown rapid thermal annealing (RTA) was carried out immediately at 600 °C for 1 min in vacuum, N_2_, and O_2_ ambients, respectively. The film thickness was measured by Woollam M2000D spectroscopic ellipsometry (SE). The microstructures of the gate insulators (LaAlO_3_ dielectric) were observed by cross-sectional high resolution transmission electron microscopy (HRTEM) performed with the [100] direction [12] of the Si substrate. The chemical composition of the fabricated films was examined by time of flight secondary ion mass spectrometry (TOF-SIMS). The band structures of the films were examined by the X-ray photoelectron spectroscopy (XPS) measurements. All the wafers were etched by Ar^+^ for 10 s (0.26 nm/s) to remove the impurities on the film surface. In this experiment, the 10-nm LaAlO_3_ film was used to obtain the XPS spectra for thick amorphous LaAlO_3_, and the 4-nm LaAlO_3_/Si structure was thin enough to obtain XPS spectra from both the LaAlO_3_ film and the underlying silicon substrate. The electrical properties of the 4-nm LaAlO_3_ films were measured using a metal-insulator semiconductor (MIS) capacitor structure. The MIS capacitors were fabricated by magnetron sputtering 150 nm Pt on the surface of the wafers through a shadow mask (metal gate with a diameter of 300 μm). The electrical properties, including capacitance-voltage (*C-V*), conductance-voltage (*G-V*), and leakage current density-voltage (*J-V*) characteristics, were measured using an Agilent B1500A analyzer.

For simplicity, the as-grown and annealed films in vacuum, N_2_, and O_2_ ambients were assigned as S1, S2, S3, and S4, respectively.

## Results and Discussion

**Fig. 1:**
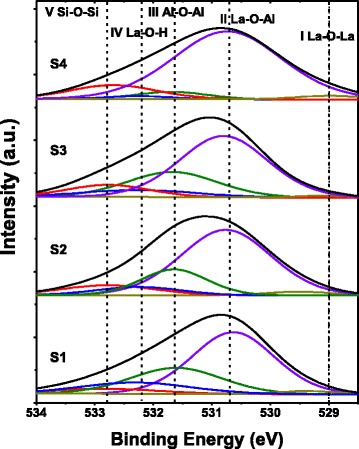
O 1*s* XPS spectra of ~4-nm LaAlO_3_ films S1 ~ S4

As shown in Fig. 1, O 1*s* XPS spectrums for the ~4-nm as-grown and annealed LaAlO_3_ films in vacuum, N_2_, and O_2_ ambients were analyzed to investigate the chemical bonding states near the interface between the LaAlO_3_ films and Si substrate. The peak position of C *1s* at 284.6 eV was used as the calibration reference. The O 1*s* core level spectra consists of five peaks, which are approximately at 529.0 eV (I), 530.7 eV (II), 531.6 eV (III), 532.2 eV (IV), and 532.8 eV (V). These peaks correspond to the chemical bonds of La–O–La, La–O–Al, Al–O–Al, La–O–H, and Si–O–Si, respectively [13, 14]. Among the five peaks, peak I and peak III come from chemical products La_2_O_3_ and Al_2_O_3_ in the deposition process. The high temperature annealing process promotes the fracture and recombination of chemical bonds, as a result, the amount of Al–O–Al decreases while the amount of La–O–Al increases. After the annealing treatments, slight variation for the La–O–La signal was observed since few La–O–La bonds were formed in the deposition process due to the high formation enthalpy of La_2_O_3_ [15]. Peak IV, related to La(OH)_*x*_, may come from the hygroscopicity of the La_2_O_3_ [16]. After high temperature annealing treatments, significant decrease in the intensity of peak IV could be observed, indicating the reduction of hydroxyl groups [17]. It is found that the intensity of Peak V shows a more obvious increase when the annealing treatment was carried out in O_2_ ambient compared with that in N_2_ or vacuum ambient, indicating that more Si–O–Si bonds were formed during the RTA process in O_2_ ambient. The Si–O–Si bonds are considered to come from SiO_*x*_, which is a main component of IL between the LaAlO_3_ film and silicon substrate [18]. So, it can be concluded that the formation of IL was enhanced during the RTA process, especially in O_2_ ambient.

**Fig. 2:**
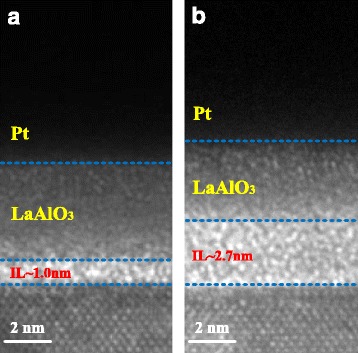
Cross-sectional HRTEM images of ~4-nm fabricated LaAlO_3_ samples. **a** S1. **b** S4

**Fig. 3:**
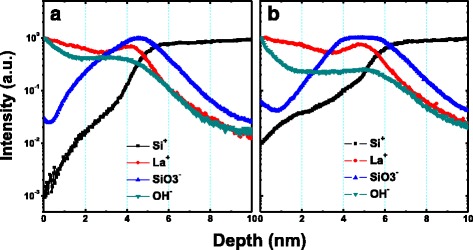
TOF-SIMS depth profiles of ~4-nm fabricated LaAlO_3_ samples. **a** S1. **b** S4

To further investigate the structural information at the dielectric/Si interface, cross-sectional HRTEM analyses for S1 and S4 are shown in Fig. 2. Both S1 and S4 exhibit an amorphous structure as no nanometer-sized crystal or long-range ordered crystal region was observed [19]. Compared with Fig. 2a, a much thicker amorphous transition region about ~2.7 nm between the deposited film and Si substrate is observed in Fig. 2b, indicating a much thicker IL formation during the annealing process in O_2_ ambient. We attribute this difference of IL formation to the RTA-induced interdiffusion of LaAlO_3_ films and silicon substrates. In order to address the evolution of the chemical composition at the LaAlO_3_/Si interface and within the LaAlO_3_ films, TOF-SIMS depth profiles of Si^+^, La^+^, SiO_3_^−^, and OH^−^ clusters were acquired on S1 and S4, as shown in Fig. 3. The intensity of the signals was dealt with normalization method, and depth values were calibrated by HRTEM results. As shown in the depth profiles of Si^+^ and La^+^, during the annealing process, substrate Si atoms diffuse into the upper LaAlO_3_ film, and the diffusion of La atoms in the opposite direction occurs simultaneously. HRTEM analysis reveals the existence of a thicker IL in S4, and now this result can be further confirmed from the intensity of SiO_3_^−^ signals which suggest the extra presence of a SiO_*x*_-like component coexisting with the La-based profile (La^+^) in the region at the nanolaminate/substrate interface for S4. Besides, compared with S1, the OH^−^ profile is reduced after annealing treatment in O_2_ ambient, in good agreement with the XPS results.

**Fig. 4:**
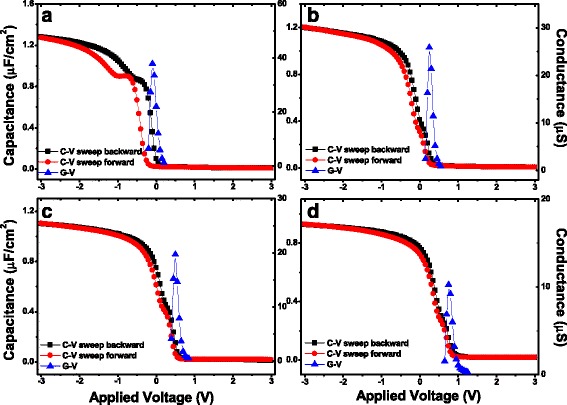
*C-V* and *G-V* characteristics for the fabricated MIS capacitors using 4-nm S1 ~ S4 as insulators. **a** S1. **b** S2. **c** S3. **d** S4. The capacitors were measured at the frequency of 100 kHz

Figure 4 shows the *C-V* and *G-V* characteristics of the fabricated MIS capacitors using the as-grown and annealed LaAlO_3_ films as insulators. *C-V* characteristics were obtained by sweeping forward (bias from negative to positive) and backward (bias from positive to negative) at the frequency of 100 kHz. *G-V* curves were obtained simultaneously with the *C-V* curves measured with applied voltage sweeping from positive to negative. The accumulation capacitance values of the MIS capacitors using the fabricated LaAlO_3_ films S1 ~ S4 as insulators were obtained to be 1.28, 1.20, 1.10, and 0.93 μF/cm^2^, respectively. The annealing treatment of LaAlO_3_ films in different ambients results in varying degrees of decrease in accumulation capacitance. In accordance with the XPS results shown in Fig. 1 and TOF-SIMS results shown in Fig. 3, such decreases in the accumulation capacitance are attributed to the formation of lower dielectric constant ILs, which primarily consist of SiO_*x*_-like component and La-silicate, due to the interdiffusion of LaAlO_3_ films and silicon substrates during the RTA treatment [20].

The flat band voltages (*V*_FB_) of the capacitors were extracted from the simulation software named Hauser NCSU CVC program taking into account of quantum-mechanical effects [21]. Considering the work function difference between the p-type Si substrate and Pt electrode, the ideal *V*_FB_ should be 0.73 V. However, the actual *V*_FB_ swept backward for the as-grown LaAlO_3_ film is 0.01 V, indicating the existence of effective positive oxide charges in the LaAlO_3_ film, which may be attributed to the existence of positive fixed oxide charges and oxide-trapped charges. Compared with the as-grown LaAlO_3_ film, positive *V*_FB_ shifts for the LaAlO_3_ films annealed at 600 °C in vacuum, N_2_, and O_2_ ambients were observed to be 0.06, 0.33, and 0.51 V, respectively, revealing the reduction of positive oxide charges during the RTA treatments [22]. Assuming the two-dimensional distribution of traps in the vicinity of the interface contributing to the film capacitance, we investigated the charge trapping behavior through the *C-V* hysteresis characteristics. The trapped charge density (*N*_ot_) can be estimated following the equations [23, 24]:

1 $$ {C}_{\mathrm{ox}}={C}_{\mathrm{ac}}\left[1+\left(\frac{G_{\mathrm{ac}}}{\omega {C}_{\mathrm{ac}}}\right)\right] $$

2 $$ {N}_{\mathrm{ot}}=\frac{\varDelta {V}_{\mathrm{FB}}{C}_{\mathrm{ox}}}{qA} $$

Where *C*_ox_ is the gate oxide capacitance, *C*_ac_ is the measured accumulation capacitance, *G*_ac_ is the conductance in accumulation region, *q* is the electron charge (1.602 × 10^19^ C), *A* is the electrode area, and *ω* is the angular frequency. The hysteresis width (Δ*V*_FB_) of S1 ~ S4 were extracted to be 299, 135, 122, and 72 mV, separately. Thus, using Eqs. (1) and (2), the *N*_ot_ values of S1 ~ S4 were determined to be 2.47 × 10^12^, 1.03 × 10^12^, 8.47 × 10^11^, and 4.20 × 10^11^ cm^−2^, respectively. As expected, a visible decrease in *N*_ot_ could be observed after annealing treatments, indicating that the reduction of the oxide trapped charges, which may be attributed to the existence of oxygen vacancies, should be one of the causes leading to the positive shifts of *V*_FB_. In addition, the larger decrease in the magnitude of *N*_ot_ for the LaAlO_3_ film annealed in O_2_ ambient may be owing to the further reduction of oxygen vacancies during the oxygen atmosphere annealing process [25].

Moreover, as shown in Fig. 4, varying degrees of humps in the *C-V* curves could be observed, which may be caused by the existence of interfacial traps [26, 27]. Compared with Fig. 4a, it can be seen that the humps were reduced after the annealing treatments, especially in O_2_ ambient. Considering this, the values of *D*_it_ for the fabricated MIS capacitors extracted from the Hill-Coleman single-frequency approximation were discussed, and the results are shown in Table 1. The *D*_it_ values for the fabricated MIS capacitors using S1 ~ S4 as insulator are about 9.65 × 10^12^, 5.12 × 10^12^, 4.29 × 10^12^, and 2.50 × 10^12^ eV^−1^cm^−2^, respectively. After the annealing treatments in different ambients, varying degrees of decrease in the values of *D*_it_ are observed, agreeing with the variation trend of the humps in the *C-V* curves. This phenomenon can be attributed to the decrease of defects and dangling bonds near the interface during the RTA process [28, 29].

**Table 1:** Various parameters for the fabricated MIS capacitors using S1 ~ S4 as insulators

Sample	*C*_ox_ (μF/cm^2^)	*V*_FB_ (V) backward	Δ*V*_FB_ (mV)	*N*_ot_ (cm^−2^)	*D*_it_ (eV^−1^cm^−2^)	VBO (eV)
S1	1.32	0.01	299	2.47 × 10^12^	9.65 × 10^12^	3.24
S2	1.23	0.07	135	1.03 × 10^12^	5.12 × 10^12^	3.36
S3	1.12	0.34	122	8.47 × 10^11^	4.29 × 10^12^	3.46
S4	0.94	0.52	72	4.20 × 10^11^	2.50 × 10^12^	3.55

**Fig. 5:**
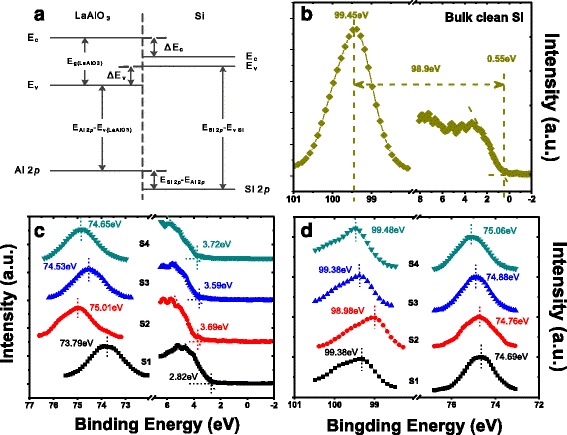
Band alignments of LaAlO_3_/Si structures. **a** Schematic of band energy alignment diagram for a LaAlO_3_/Si structure; XPS core level spectra of **b** Si 2*p* and valence band for bulk clean silicon, **c** Al 2*p* and valence band for 10-nm LaAlO_3_ films, and **d** Si 2*p* and Al 2*p* for 4-nm LaAlO_3_ films on p-Si(100)

To further investigate the interfacial properties between the LaAlO_3_ films and Si substrate, the VBOs of LaAlO_3_/Si structures were analyzed by XPS measurements. The VBOs of LaAlO_3_ films relative to Si substrate were determined by a core level photoemission-based method similar to that of Kraut et al [30, 31] as illustrated in Fig. 5a. Accordingly, the VBO is given by Eq. (3):

3 $$ \varDelta {E}_{\mathrm{v}}={\left({E}_{\mathrm{Si}\ 2 p}-{E}_{\mathrm{V}}\right)}_{\mathrm{Si}}-{\left({E}_{\mathrm{Al}\ 2 p}-{E}_V\right)}_{{\mathrm{Thick}\ \mathrm{LaAlO}}_3}-{\left({E}_{\mathrm{Si}\ 2 p}-{E}_{\mathrm{Al}\ 2 p}\right)}_{{\mathrm{LaAlO}}_3/\mathrm{Si}} $$

Where E_Si 2*p*_ is the binding energy of Si 2*p* shallow core level and E_Al 2*p*_ is the binding energy of Al 2*p* shallow core level. Valence band maximum (E_v_) is the binding energy corresponding to the top of the valence band (VB) for Si and LaAlO_3_, respectively. The positions of the E_v_ for both Si and dielectrics were determined by linearly extrapolating the segment of maximum negative slope to the background level [32].

Figure 5b, c shows the shallow core-level and VB spectra for bulk clean p-type Si(100) and thick 10-nm LaAlO_3_ films, while Fig. 5d shows the shallow core-level spectrums for 4-nm LaAlO_3_/Si structures. The energy difference for bulk p-type Si (100) between the XPS spectra of Si 2*p* and E_v_ was determined to be 98.9 ± 0.05 eV. Therefore, according to Eq. (3), the VBOs of as-grown and annealed LaAlO_3_ films in vacuum, N_2_, and O_2_ ambients relative to p-type Si substrate were measured to be 3.24 ± 0.1, 3.36 ± 0.1, 3.46 ± 0.1, and 3.55 ± 0.1 eV, respectively. It is found that the VBO values of the LaAlO_3_ films after annealing are obviously larger than that of the as-grown LaAlO_3_ film, and the largest VBO value was obtained in the O_2_ case. The augment of the VBO values after annealing treatments is believed to benefit from the formation of SiO_*x*_-like IL, which has much larger band offsets relative to silicon than that of LaAlO_3_.

**Fig. 6:**
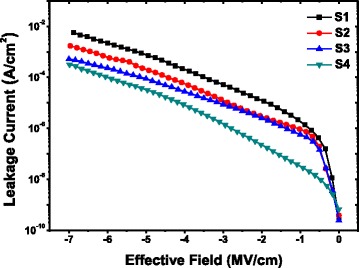
*J-V* characteristic of the fabricated MIS capacitors using 4-nm S1 ~ S4 as insulators

Figure 6 displays the leakage current density as a function of the applied electrical field of the films with the Pt/4-nm LaAlO_3_/p-type Si capacitor structures. The leakage current density for the as-grown LaAlO_3_ film was determined to be ~7.14 × 10^−4^ A/cm^2^ at the applied electrical field of −5 MV/cm. After being annealed, at the same applied electrical field, the leakage current density values of the fabricated MIS capacitors using LaAlO_3_ films annealed in vacuum, N_2_, and O_2_ ambients as insulators were measured to be ~1.86 × 10^−4^, ~8.78 × 10^−5^, ~3.18 × 10^−5^ A/cm^2^, respectively. Significant decrease in the gate leakage current was observed after being annealed, especially for the O_2_ case, in which a decrease of more than one order of magnitude was obtained. Such a decrease of leakage current density may be primarily attributed to the change of valence band offsets at the nanolaminate/Si interface during the high temperature annealing process. It has been reported that the gate leakage current for high-k dielectric depends exponentially on potential barriers, which vary with band offsets [33]. As mentioned above, among the as-grown and annealed samples in vacuum, N_2_, and O_2_ ambients, the largest VBO value was obtained in O_2_-annealed LaAlO_3_/Si structure, providing an effective potential barrier to weaken the tunneling effect of electrons and holes in the MIS capacitor, resulting in lowest gate leakage current. In addition, the annealing treatment in O_2_ ambient seems to serve as a most effective way to reduce oxygen vacancies, which may also give an explanation to the significant decrease of gate leakage current in S4.

## Conclusions

In this paper, the effects of different annealing ambients on the physical and electrical properties of LaAlO_3_ films grown by ALD were analyzed. It was found that the amount of hydroxyl groups decreased after annealing treatments. In addition, ILs are formed after annealing treatments, resulting in the decrease of accumulation capacitance values for LaAlO_3_ films, especially in O_2_ ambient. Compared with the ideal *V*_FB_, the actual *V*_FB_ value for the as-grown LaAlO_3_ dielectric was negatively shifted, indicating the existence of positive oxide charges. After RTA treatments in different ambients, oxygen vacancies and defects were reduced, resulting in positive *V*_FB_ shifts in varying degrees. Significant decrease in the leakage current density was found when the LaAlO_3_ films were annealing treated, especially for the LaAlO_3_ film annealed in O_2_ ambient, in which a decrease of more than one order of magnitude was found. Such a decrease in the leakage current density may be primarily attributed to the larger values of valence band offsets and the reduction of oxygen vacancies near the LaAlO_3_/Si interface.

## Abbreviations

ALD: Atomic layer deposition
CMOS: Complementary metal oxide semiconductor
C-V: Capacitance-voltage
*D*_it_: Interface trap density
EOT: Equivalent oxide thickness
E_v_: Valence band maximum
G-V: Conductance-voltage
HRTEM: High resolution transmission electron microscopy
IL: Interfacial layer
J-V: Leakage current density-voltage
MIS: Metal-insulator semiconductor
*N*_ot_: Trapped charge density
PDA: Post-deposition annealing
RTA: Rapid thermal annealing
SE: Spectroscopic ellipsometry
TOF-SIMS: Time of flight secondary ion mass spectrometry
VB: Valence band
VBO: Valence band offset
*V*_FB_: Flat band voltage
XPS: X-ray photoelectron spectroscopy
Δ*V*_FB_: Hysteresis width of *V*_FB_
